# The ScotCap registry: An evaluation of 1000 colon capsule endoscopy procedures carried out in Scotland

**DOI:** 10.1111/codi.17271

**Published:** 2024-12-29

**Authors:** C. MacLeod, N. Rajapaksha, C. Brown, J. Hudson, Z. Asif, A. J. M. Watson, the ScotCap clinical leads, C Mowat, P Cartlidge, R Gratton, J Winters, Z Bell, C Ray, F Maxwell, A McKinley, C Noble, P Collins, L Wilson, N Cruikshank, P Hendry, J Fletcher, B Weber

**Affiliations:** ^1^ Department of Surgery Aberdeen Royal Infirmary Aberdeen UK; ^2^ Institute of Applied Health Sciences, University of Aberdeen Aberdeen UK; ^3^ Public Health Scotland Edinburgh UK; ^4^ Health Services Research Unit University of Aberdeen, Health Sciences Building Aberdeen UK; ^5^ Department of Surgery University Hospital Hairmyres East Kilbride UK; ^6^ Department of Surgery Raigmore Hospital Inverness UK

**Keywords:** colon capsule endoscopy, colonoscopy, colorectal cancer

## Abstract

**Aim:**

Colon capsule endoscopy (CCE) has been deployed in Scotland as an alternative to colonoscopy. The aim of this work was to report the outcomes of patients undergoing CCE in Scotland from the national CCE registry.

**Method:**

Patients undergoing CCE in NHS Scotland between 1 August 2020 and 1 August 2021 were included in this study. Symptomatic and surveillance patients were vetted to undergo CCE by clinicians in four NHS Scotland health boards. Patient data were collected prospectively by an eHealth system managing CCE referral and report sign off. CCE procedural data were collected by a managed service carrying out CCE procedures. Data from both sources were compiled in the national CCE registry managed by Public Health Scotland.

**Results:**

One thousand and eighty‐seven patients were included in this study. The median age of patients was 60 years and 58.3% of patients were female. A haemoglobin(Hb) count was available for 657 (60.4%) patients and the mean value was 139 g/L. Faecal immunochemical test results were available for 762 (70.1%) patients, and of those with a test result 79.0% had a result ≥10 μgHb/g. The rates of complete test, adequate bowel preparation and successful test were 57.0%, 59.2% and 56.9%, respectively. Following CCE, no further test, colonoscopy, flexible sigmoidoscopy or gastroscopy were required for 42.0%, 34.1%, 22.7% and 2.6% of patients, respectively. Two (0.2%) patients experienced capsule retention with obstruction.

**Conclusion:**

These are the first reported outcomes from the ScotCap registry; they demonstrate that CCE is a safe alternative to colonoscopy and a significant proportion of patients avoid colonoscopy when CCE is implemented nationally.

**What does this paper add to the literature?**

The ScotCap registry has collected national‐level data for patients undergoing colon capsule endoscopy (CCE) in Scotland. The data from the registry add to a growing body of literature demonstrating that CCE is a safe test, and a significant proportion of patients undergoing CCE avoid colonoscopy.

## INTRODUCTION

Colon capsule endoscopy (CCE) is a noninvasive diagnostic alternative to colonoscopy [1]. The capsule captures images of the lining of the gastrointestinal (GI) tract as it travels through it; these are compiled into a video for interpretation. Pathology such as polyps, colorectal cancer (CRC) and colonic inflammation can be detected by CCE. The reported diagnostic accuracy of CCE for the detection of colonic neoplasia is comparable to colonoscopy, and CCE has a greater sensitivity for the detection of polyps compared with CT colonogram (CTC) [2, 3, 4]. Furthermore, CCE is a safe, well‐tolerated procedure and, due to its noninvasive and painless nature, is equally preferred to colonoscopy by patients [5].

The ScotCap programme, a multicentre clinical evaluation, has highlighted the benefits of using CCE in clinical practice when it is embedded into standard lower GI diagnostic pathways [6]. In 2022 we reported results from one of the largest published cohorts of patients who had undergone CCE for new lower GI symptoms and for colonic surveillance. Symptomatic patients were found to be better suited for CCE than surveillance patients as they were more likely to require no further test (37.3% vs. 27.5%). In addition, the evaluation reported improved triaging of endoscopic tests as patients could be appropriately selected for endoscopic therapy based on CCE findings. However, the rate of complete test (visualization of the whole colon and rectum) experienced in the evaluation was suboptimal in both cohorts and failed to match the performance of colonoscopy [7].

In the United Kingdom (UK), the use of CCE is expanding to meet ongoing shortfalls in colonoscopy capacity [8, 9, 10, 11]. As the use of CCE continues to increase there is a need to continually evaluate the clinical outcomes and safety profile of the diagnostic test. The IDEAL framework provides guidance for the evaluation of surgical therapy innovation and large‐scale surveillance of outcomes. Similar recommendations from the ‘First Do No Harm’ report also advocate for the establishment of a central patient‐identifiable database to research and audit outcomes [12, 13]. Although both these recommendations are primarily for surgical therapy or the use of implants, a national CCE registry was established to collect and monitor the outcomes of all patients undergoing CCE in Scotland. In this prospective study, which follows the initial ScotCap evaluation, we report the outcomes of the first 1000 patients captured in the national CCE registry.

## METHOD

### Study participants

Patients undergoing CCE in NHS Scotland between 1 August 2020 and 1 August 2021 were included in this study. CCE procedures were carried out in four NHS Scotland health boards: NHS Highland, Greater Glasgow and Clyde, Tayside and Lanarkshire. Symptomatic patients (those with symptoms suggestive of colorectal pathology referred by primary care) were vetted to undergo CCE by clinicians in these health boards following national guidance (Appendix S1). Symptomatic patients requiring lower GI investigation were predominantly chosen to undergo CCE rather than surveillance patients. Surveillance patients could also undergo CCE if a clinician assessed the patient as appropriate on a patient‐by‐patient basis provided they did not meet any of the exclusion criteria for the procedure. Adult patients (>18 years of age) with lower GI symptoms and a faecal haemoglobin (f‐Hb) between 10 and 150 μg/g [measured by a faecal immunochemical test (FIT)] or iron‐deficiency anaemia were considered suitable to undergo CCE. Patients who were medically unfit to take full bowel preparation, had trouble swallowing, had indwelling electromedical devices or insulin‐dependent diabetes mellitus were excluded from undergoing CCE. In addition, patients whose main symptom was constipation were also deemed to be unsuitable to undergo CCE due to concerns about slow transit of the capsule and obtaining an incomplete test. Scottish bowel screening patients were also excluded from the study. Patients identified by local health board clinicians as suitable for CCE were referred to a managed service to undergo the test.

### Procedures

The PillCam™ COLON 2 (Medtronic) was used for all procedures in this study. CCE procedures were carried out by trained nursing staff in community healthcare centres or secondary‐care hospitals in each health board. A procedural protocol (Appendix S2) was used to ensure CCE tests were carried out in the same manner across Scotland. Pretest dietary recommendations and the bowel preparation and booster regimen (Appendix S3) used were also consistent across all health boards for the study duration. CCE procedures were interpretated and reported by two NHS Scotland consultant gastroenterologists who had undergone training in CCE reading. CCE reports were reviewed by the referring clinician in each health board to decide if further investigation or management was required. In general, the European Society of Gastrointestinal Endoscopy guidelines were used to decide on the management of polyps found by CCE [14]. Follow‐up tests were carried out in line with local standard care.

A complete test was defined as a procedure that visualized the whole colon and rectum. The bowel preparation for each colonic segment (right, transverse colon, left colon including rectum) was rated using the Leighton–Rex bowel preparation scale [15]. The bowel preparation was deemed to be adequate if all colonic segments were rated as at least ‘fair’ and the overall quality was judged to be acceptable by the CCE reader. A CCE was defined as ‘successful’ if the test was complete and the bowel preparation was adequate.

### Data collection

Data used in this study were collated in the ScotCap registry, a national CCE registry managed by Public Health Scotland (PHS). Registry data were compiled from three different sources in REDCap (Research Electronic Data Capture) and linked using each patient's unique Community Health Index (CHI) number [16]. Patient data were collected prospectively by an eHealth system [Endoscopy Management System (EMS), Medilogik] managing CCE referral (from secondary care to the managed service) and report sign off. Demographic data, urgency of referral [urgent suspect cancer (USC), urgent or routine as dictated by the primary care referral] and pre‐CCE test investigation results (such as Hb count and f‐Hb) were entered by the referring clinician at the point of CCE referral into the EMS. Follow‐up test requirement (e.g. colonoscopy) and adverse CCE events were recorded by the clinician reviewing and signing off the CCE report in the EMS. CCE procedural data, including bowel preparation rating, completeness of the test and pathology found, were collected prospectively by the ScotCap managed service in an automated fashion and uploaded into REDCap. Follow‐up test (colonoscopy and flexible sigmoidoscopy) findings were collected by clinicians retrospectively in local health boards and manually entered into REDCap.

Public Benefit and Privacy Panel (PBPP) approval was obtained for the collection and publication of the data from the ScotCap registry. This study was carried out as a service evaluation and further research ethical approval was not required. The protocol used for this study is available online (https://osf.io/puk9g/files/osfstorage/65d781630d9acb004cc2eb9d).

### Outcomes

The primary outcome was the proportion of patients requiring a further test (nil, colonoscopy, flexible sigmoidoscopy, CTC, surgical intervention, clinical follow‐up and other) following CCE. Secondary outcome measures were the rate of complete testing, adequate bowel preparation and successful test, pathology (polyp, inflammation or CRC) detected by CCE, and adverse events.

### Statistical analysis

Polyp matching analysis was conducted using previously published methodology [3, 6, 17]. Patients who underwent a follow‐up colonoscopy and had a CCE procedure that was complete with adequate bowel preparation were included for polyp matching. A polyp was considered a true positive if it met two criteria: the polyp reported by CCE had to be found at colonoscopy in the same or adjacent colonic segment as reported by CCE, and the size of the polyp reported by CCE ±50% overlapped with the size found at colonoscopy ±50%. Polyps found at colonoscopy but not reported by CCE were regarded as false negatives. Polyps reported by CCE but not found at colonoscopy were considered false positives. Polyps <6 mm were not considered for polyp matching purposes. The sensitivity of CCE for the detection of polyps ≥6 mm and ≥ 10 mm was calculated on a per polyp and per patient basis in the respective size range. The specificity of CCE for the detection of polyps was not calculated, since most patients undergoing CCE in previous studies were due to pathology and therefore there would be only a small number of patients who would be considered true negatives. Data validation was carried out prior to analysis. Data were analysed descriptively and using R (v. 4.1.2).

## RESULTS

During the study period, 1 August 2020 to 31 July 2021, 1087 patients underwent CCE. Data collection concluded on 1 August 2023. The median age of all patients was 60 years and 58.3% were female (Table 1). Most patients (95.9%) were symptomatic, and the remainder underwent CCE for surveillance reasons. The presenting symptoms most recorded were ‘other’ and ‘abdominal distress/pain’. Hb and FIT results were recorded for 60.4% and 70.1% of patients, respectively. The mean Hb value was 139.2 g/L. Of those with a FIT result, 20.7%, 78.5%, 0.5% and 0.3% had a FIT result <10 μgHb/g, 10–150 μgHb/g, 151–399 μgHb/g and ≥400 μgHb/g, respectively. The most common surveillance reason for undergoing CCE was a family history of CRC. The median time between referral for CCE and report being received was 54 days.

**TABLE 1:** Baseline characteristics.

Characteristic	
No. of patients undergoing CCE	1087
Age (years), median (SD)	60 (±13.53)
Gender	
Female	634 (58.3)
Male	453 (41.7)
Patient type	
Symptomatic	1042 (95.9)
Surveillance	45 (4.1)
Urgency of referral	
Urgent suspected cancer^a^	315 (29.0)
Urgent	436 (40.1)
Routine	336 (30.9)
Symptomatic referral reasons^b^	
Abdominal distress/pain	334 (30.7)
Anaemia	81 (7.5)
Alternating bowel habit	134 (12.3)
Constipation	51 (4.7)
Diarrhoea	121 (11.1)
Melaena	7 (0.6)
Rectal bleeding	251 (23.1)
Weight loss	78 (7.2)
Other	510 (46.9)
Surveillance referral reasons^b^	
Family history of colorectal cancer	44 (4.0)
Inflammatory bowel disease assessment	1 (0.1)
Incomplete colonoscopy	3 (0.3)
Polyp surveillance	1 (0.1)
Hb (g/L), mean (SD); *n*	139.2 (±38.88); 657
FIT (μgHb/g)	
<10	158 (14.5)
10–150	598 (55.0)
151–399	4 (0.4)
≥400	2 (0.2)
Missing	325 (29.9)

*Note*: Values are *n* (%) unless otherwise stated.

Abbreviations: CCE, colon capsule endoscopy; FIT, faecal immunochemical test; Hb, haemoglobin.

^a^ Equivalent to NHS England 2‐week‐wait pathway.

^b^ More than one symptom/reason could be selected.

The proportion of patients obtaining a complete test, adequate bowel preparation and a successful test was 57%, 59.2% and 56.9%, respectively (Table 2). Following CCE, 58% of patients required a further test and 42% of patients required no further test (Table 3). The proportion of patients who required a colonoscopy, flexible sigmoidoscopy and upper GI endoscopy following CCE was 34.1%, 22.7% and 2.6%, respectively. Two serious adverse events were recorded during the study period, both were instances of capsule retention with obstruction which required surgical intervention. Additionally, five patients were recorded as having complications related to bowel preparation.

**TABLE 2:** Completion rates, bowel preparation rating and pathology detected by colon capsule endoscopy (CCE) (*N* = 1087).

Complete test^a^	620 (57.0)
If incomplete‐ gastrointestinal tract visualized to:	
Caecum	374 (60.3)
Right colon	364 (33.5)
Transverse colon	324 (29.8)
Left colon	225 (20.7)
Rectum	25 (2.3)
Overall adequate bowel preparation^b^	644 (59.2)
Adequate bowel preparation by segment	
Caecum	870 (80.0)
Right colon	864 (79.5)
Transverse colon	846 (77.8)
Left colon	756 (69.5)
Rectum	557 (51.2)
Successful test rate^c^	619 (56.9)
Number of polyps found per patient	
0	403 (37.1)
1–4	492 (45.3)
5–10	162 (14.9)
>10	30 (2.8)
Largest polyp found^d^	
≤6 mm	205 (30.0)
7‐9 mm	185 (27.0)
≥10 mm	294 (43.0)
Polyp detection rate	
Any size	684 (62.9)
≥6 mm	556 (51.1)
≥10 mm	294 (27.0)
Colorectal cancer	13 (1.2)
Inflammatory bowel disease	10 (0.9)

*Note*: Values are *n* (%).

^a^ Visualization of the whole colon and rectum.

^b^ At least ‘fair’ in all segments and determined as adequate overall by CCE reader.

^c^ A complete CCE with adequate bowel preparation.

^d^ Percentages as a proportion of patients with a polyp found.

**TABLE 3:** Patient outcomes (N = 1087).

Outcome	
No further test	457 (42.0)
Colonoscopy	371 (34.1)
Flexible sigmoidoscopy	247 (22.7)
CT colonogram	0 (0)
Upper gastrointestinal endoscopy	28 (2.6)
Capsule retention with obstruction	2 (0.2)
Bowel prep related	5 (0.5)

*Note*: Values are *n* (%).

The results of the polyp matching analysis are reported in Table 4. For the purposes of polyp matching analysis, 184 patients had a complete CCE with adequate bowel preparation and had undergone a colonoscopy before data collection was concluded. The calculated sensitivity of CCE for the detection of polyps ≥6 mm and ≥ 10 mm on a per polyp basis was 97.1% and 95.8%, respectively. On a per patient basis, the calculated sensitivity of CCE for the detection polyps ≥6 mm and ≥ 10 mm was 95.2% and 94.9%, respectively. Thirteen patients were reported to have CRC by CCE, and all patients' CRCs were subsequently detected by follow‐up colonoscopy.

**TABLE 4:** Polyp matching analysis.

	Polyp ≥ 6 mm	Polyp ≥ 10 mm
Per polyp		
True‐positive polyps reported by CCE	237	136
False‐positive polyps reported by CCE	233	68
False‐negative polyps reported by CCE	7	6
Per patient		
True‐positive patients reported by CCE	120	94
False‐positive patients reported by CCE	91	41
False‐negative patients reported by CCE	6	5

*Note*: values are *n*.

Abbreviation: CCE, colon capsule endoscopy.

## DISCUSSION

In this registry study, 42% of patients required no further test following CCE, and 34.1% and 22.7% required a colonoscopy and flexible sigmoidoscopy, respectively. While there has been a modest reduction in the proportion of patients requiring no further test in this study compared with the symptomatic cohort in the ScotCap evaluation (42% vs. 37%), over half the patients undergoing CCE required further endoscopy tests [6]. The reporting of the clinical outcomes described in this study has been facilitated by the establishment of the ScotCap registry, which was an important part of the implementation of CCE in Scotland. Importantly, we have been able to capture safety profile data which have shown that the rate of capsule retention with obstruction (the most serious adverse event associated with CCE) is 0.2% in this study, consistent with other published rates [18].

There remain significant challenges in meeting the demand for lower GI investigation in NHS Scotland. Colonoscopy waiting times continue to fail to meet national targets and additional capacity is needed to manage the backlog [10]. The impact that CCE has had on waiting times for colonoscopy is unclear. Lower GI diagnostic capacity has been increased with the roll‐out of CCE; however, the direct impact of CCE on waiting times is difficult to measure. Published CCE studies have also shown that CCE can improve triaging for subsequent endoscopy tests and save endoscopy time, even when taking follow‐up test requirements into account [19]. Given these benefits for diagnostic capacity, CCE should continue to be considered as an alternative test to colonoscopy in lower GI diagnostic pathways.

Reassuringly, CCE identified all CRC lesions detected at subsequent endoscopy in this study. To date, CCE has been shown to have a high sensitivity for the detection of CRC and reports of missed CRC by CCE are rare [4, 20]. No long‐term follow‐up data are available for patients included in this study. However, the ScotCap registry has been developed to allow linkage to national cancer registries to ensure that the post‐CCE interval CRC rate (the proportion of patients with CRC diagnosed after a normal test), which is yet to be reported, can be calculated. The 3‐year interval CRC rate for colonoscopy and CTC is well established (7.4% and 4.4%, respectively), and publication of the interval CRC rate for CCE will be an important metric to allow direct comparison with these established tests [21, 22].

The primary aim of this study was to evaluate the clinical outcomes for patients undergoing CCE rather than the diagnostic accuracy of CCE, which is well reported [4]. However, polyp matching analysis was conducted for patients who had a complete CCE with adequate bowel preparation and underwent follow‐up colonoscopy. Our analysis showed comparable sensitivity for polyp detection with the most recently published meta‐analysis of CCE diagnostic accuracy. Most patients had a polyp detected by CCE (62.9%) and the polyp detection rate in this study was 51.1% for polyps ≥6 mm. These findings highlight that the issue of using CCE in clinical practice is not related to missing pathology but that a high proportion of patients who have polyps detected by CCE need a subsequent follow‐up test. In addition, not all polyps detected by CCE will be found by colonoscopy, as demonstrated by the number of false‐positive polyps in this study. Further guidance is therefore needed for clinicians when deciding on follow‐up for patients, particularly those with small or intermediate sized polyps, as these have a low risk of progression and their removal may not be of overall benefit to the patient [23].

National level CCE outcome data, similar to that of this study, have been published from France and Germany. In France, Benech et al. conducted the ONECC study and recruited 689 patients to undergo CCE between 2011 and 2016 [24]. Similar complete test and adequate bowel preparation rates were experienced to our study, despite the use of a different booster regimen and an older patient cohort (median age 70 vs. 60 years) in the French study. In the ONECC study a greater proportion of patients required no further test compared with ours (59.4% vs. 42%); this could be explained by the differences in patient recruitment as their indications for CCE were broader than ours. Data from a smaller study (*n* = 161) have been published from a German national CCE registry [25]. The DEKOR study has published outcomes for 161 patients undergoing CCE between 2010 and 2015, predominately for investigation of symptoms (47.1%) and CRC screening (35.9%). Further diagnostic tests were required for 53.6%, with the remaining patients (47.6%) requiring no further test. The ScotCap registry data described in our study add to these real‐word data and highlight the variability in the need for tests between patient populations, the reasons for which should be explored further.

The proportion of patients with CRC in this study was low (1.2%). This could be explained by the low FIT result range used for inclusion when referring for CCE. Authors of this study have previously suggested that CCE is likely to be better suited for patients with a lower f‐HB to reduce the endoscopy requirements following CCE [26]. Previously published studies have shown that the number needed to scope (the number of patients requiring a colonoscopy to diagnose one CRC) for patients who have a lower f‐Hb is high and may not be an effective use of endoscopy capacity [27]. CCE appears to best suited for these patients, allowing colonoscopy to be reserved for those patients with a higher risk of harbouring colorectal pathology as identified by their FIT result. Although most patients (78.5%) with a FIT result undergoing CCE in this evaluation had a f‐Hb between 10 and 150 μgHb/g, there is some heterogeneity to our sample as clinical acumen and local endoscopy demand influenced patient selection for CCE. Moreover, the conclusions from our study are pertinent for symptomatic and surveillance patients undergoing CCE, not patients with a positive bowel screening test. Further results from the CareForColon 2015 trial (a large, randomized control trial examining CCE outcomes for patients in the Danish CRC screening programme) are awaited which will influence the use of CCE in the bowel screening setting [28, 29].

It has not been possible in this study to report the overall cost of implementing CCE in Scotland. The economic analysis derived from data reported by the ScotCap evaluation showed that an additional per patient cost for CCE (compared with colonoscopy) was £54 in the first year of test adoption [30]. The main contributors to the cost of the CCE pathway is the cost of the capsule and follow‐up procedures. The cost of the capsule needs to be reduced to make CCE more economically attractive for healthcare service providers as the rate of further testing has not significantly reduced with wider CCE use. However, future economic analysis should consider the cost of alternative methods to generate lower GI capacity (such as private provision of colonoscopy) to support NHS Scotland endoscopy units, as they may be more costly than CCE.

One of the strengths of this study is its size. The data report outcomes from patients undergoing CCE from different health boards across Scotland and represents clinical practice that will likely be reproducible in similar clinical settings. Additionally, patients included in this study all underwent CCE using the same procedural protocol, dietary recommendations and bowel preparation and booster regimen, reducing variation in practice across health boards. There are weaknesses to our study. The data derived for this study are from a registry which, although most data have been collected prospectively, still has missing data. For example, not all patients had a FIT result or Hb count available. Further, no patients following CCE were referred for a CTC, which may be inaccurate as 2.9% of symptomatic patients in the ScotCap evaluation underwent CTC following CCE. Finally, it was not possible to capture important demographic features of patients undergoing CCE, such as ethnicity or measures of deprivation, to ensure that access to CCE is equitable. Future research should be undertaken to ensure that patients from different socio‐economic or ethnic minority groups are able to undergo CCE equally.

## CONCLUSION

In this registry study we have found that a significant proportion of patients avoided any further test following CCE (42%). Establishing a CCE registry has allowed the reporting of important clinical outcomes and will also enable future research such as the post‐test interval CRC rate. Further work is needed to evaluate the cost effectiveness of introducing CCE into a national health service, but it should continue to be considered as an alternative to colonoscopy by endoscopic units continuing to manage a shortfall in colonoscopy capacity.

## AUTHOR CONTRIBUTIONS

**C. MacLeod:** Conceptualization; methodology; writing – original draft; writing – review and editing; formal analysis; data curation. **N. Rajapaksha:** Methodology; formal analysis; project administration. **C. Brown:** Methodology; formal analysis; project administration. **J. Hudson:** Methodology; writing – review and editing. **Z. Asif:** Data curation; writing – review and editing. **A. J. M. Watson:** Conceptualization; methodology; supervision; writing – review and editing.

## FUNDING INFORMATION

No funding was received by any author to carry out work in this study.

## CONFLICT OF INTEREST STATEMENT

The authors declare that they have no conflict of interest.

## ETHICS STATEMENT

Public Benefit and Privacy Panel approval was obtained for the collection and publication of the data from the ScotCap registry. This work was a service evaluation and therefore no further research ethics approvals were required.

## PATIENT CONSENT STATEMENT

Public Benefit and Privacy Panel approval was obtained for this work, therefore individual patient consent was not required.

## COLLABORATORS

ScotCap clinical leads: C Mowat^1^, P Cartlidge^1^, R Gratton^2^, J Winters^2^, Z Bell^3^, C Ray^3^, F Maxwell^4^, A McKinley^5^, C Noble^6^, P Collins^7^, L Wilson^8^, N Cruikshank^9^, P Hendry^10^, J Fletcher^11^, B Weber^12^.

^**1**^NHS Tayside ^**2**^NHS Greater Glasgow and Clyde ^**3**^NHS Ayrshire & Arran ^**4**^NHS Lanarkshire ^**5**^NHS Grampian ^**6**^NHS Lothian ^**7**^NHS Dumfries and Galloway ^**8**^NHS Orkney ^**9**^NHS Fife ^**10**^NHS Forth Valley ^**11**^NHS Borders ^**12**^NHS Shetland.

## Supporting information


Appendices S1–S3.

## Data Availability

The datasets for this study are governed by Public Health Scotland, and due to ethical and confidentiality reasons supporting data are not available.
