# Supplementary material for: The ScotCap registry: An evaluation of 1000 colon capsule endoscopy procedures carried out in Scotland
Source: Colorectal Dis. 2024 Dec 29;27(1):e17271. doi: 10.1111/codi.17271 (PMC11683389; doi:10.1111/codi.17271)
Supplement: Supplementary file 1 — Appendices S1–S3. [file CODI-27-0-s001.docx]

**The ScotCap registry: an evaluation of 1000 colon capsule endoscopy procedures carried out in Scotland**

**Authors**

C MacLeod (0000-0001-9161-0661)^1,2^, N Rajapaksha^3^, C Brown^3^, J Hudson^4^, AJM Watson^5^ on behalf of the ScotCap clinical leads

^1^ Department of Surgery, Aberdeen Royal Infirmary, Aberdeen, UK

^2^ Institute of Applied Health Sciences, University of Aberdeen, Aberdeen, UK

^3^ Public Health Scotland, UK

^4^ Health Services Research Unit, University of Aberdeen, Health Sciences Building, Aberdeen, UK

^5^ Department of Surgery, Raigmore Hospital, Inverness, UK

**Corresponding author**

Mr Campbell MacLeod

Email – [Campbell.macleod@nhs.scot](mailto:Campbell.macleod@nhs.scot)

Address – Department of Surgery, Aberdeen Royal Infirmary, Foresterhill Road, Aberdeen, AB25 2ZN, UK

ORCID- 0000-0001-9161-0661

**Supplementary Materials - Index**

| **Appendix A - Supplementary Figure 1** |  |
| --- | --- |
| National referral guidance for CCE | *Page 2* |
| **Appendix B - Supplementary Text 1** |  |
| CCE Procedure protocol | *Page 3* |
| **Appendix C - Supplementary Table 1** |  |
| Dietary recommendations, Bowel preparation and booster regimen for CCE procedures | *Page 4* |

# **
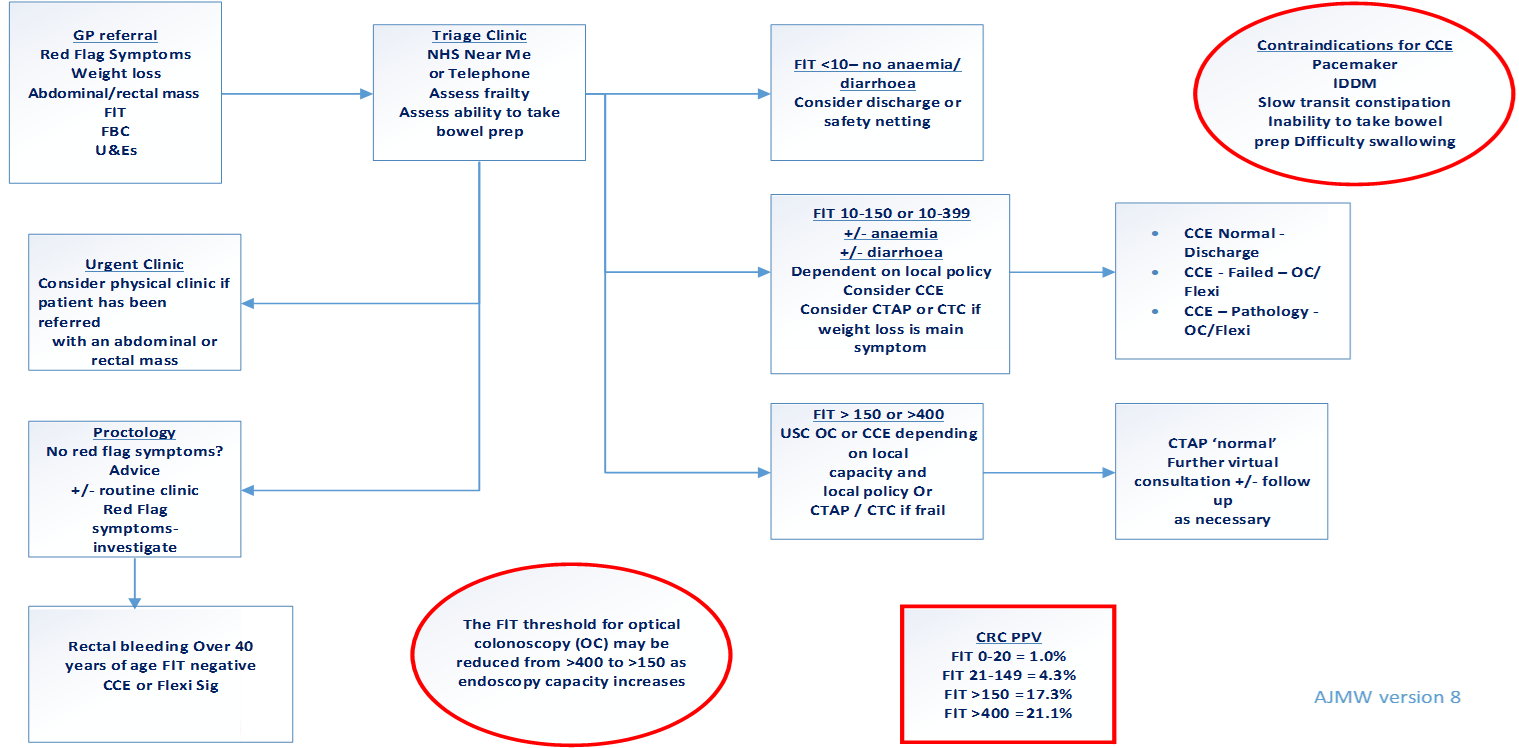
**Appendix A - Supplementary Figure 1. National referral guidance for CCE

Appendix B - Supplementary Text 1. CCE Procedure protocol

1. Patients agreeing to undergo CCE are screened by a nurse who reviews their electronic healthcare records.
2. A telephone consultation is carried out by a specialist nurse to explain the procedure in detail, confirm consent to continue with the procedure and arrange test date.
3. The bowel preparation and associated instructions are sent to the patient.
4. Support for completing the bowel preparation is provided via a telephone consultation by a nurse.
5. The patients attends their CCE appointment and the procedure is carried out by a trained specialist nurse.
6. Pre procedure checks are carried out to ensure the patient is safe to continue with the procedure and that the bowel preparation has been adequate.
7. The belt and recorder are fitted, then the capsule is swallowed by the patient.
8. The booster medication is provided to the patient with associated instructions.
9. The patient returns home to complete the procedure returning the belt and recorder the following day.
10. The CCE recording is reported using the rapid reader software (Medtronic) by an NHS Scotland gastroenterologist trained in CCE reading.

Appendix C - Supplementary Table 1. Dietary recommendations, bowel preparation and booster regimen for CCE procedures

| **Table 1. Dietary recommendations, bowel preparation and booster regimen for CCE procedures** | | |
| --- | --- | --- |
| **Day** | **Control regimen** | **Dietary recommendations** |
| 3 days before procedure | 1 sachet of Macrogol 3350 (Laxido) in 125ml of water morning and evening | Normal diet |
| 2 days before procedure | 1 sachet of Macrogol 3350 in 125ml of water morning and evening | Low residue diet |
| 1 day before procedure | 2 Litres of Polyethylene glycol solution (Klean Prep) taken over 2 hours in the evening | Clear liquid diet |
| Day of procedure | 2 Litres of Polyethylene glycol solution taken over 2 hours in the morning | Clear liquid diet |
|  | Metoclopramide hydrochloride 10mg tablet taken immediately after the colon capsule is swallowed | Return to normal diet 6 hours after capsule ingestion or once excreted. |
|  | 1 litre of Sodium picosulfate (Picolax) solution taken as indicated by the three data recorder signals |  |
|  | 1 Bisacodyl suppository (optional) to be used if the capsule has not been excreted 10h after swallowing the capsule. |  |
